# Early History of Cardiac Pacing and Defibrillation†

**Published:** 2002-01-01

**Authors:** Seymour Furman

**Affiliations:** Editor in Chief, PACE, Founder and Past President of NASPE, Principal Investigator of Electricity and Heart, The Albert Einstein College of Medicine

## Introduction

The Electricity and the Heart website [1] is intended to facilitate the collection, cataloging and presentation of historical information about technical and scientific advances in cardiac devices. Over the course of the past century as the fields of cardiac pacing and electrophysiology have evolved, the technological devices used by physicians and researchers has been a fascinating and rapidly changing portion of the history of the fields. NASPE's Oral History Project houses hundreds of devices collected over the years that illustrate the evolution from crude and simple machines to the sophisticated and advanced technological wonders that are used in the field today. The photos and descriptions of many of these devices show just how far we have come in the advancement of treatment and patient care.

## The 1930s: Hyman Pacemaker

**Figure 1:**
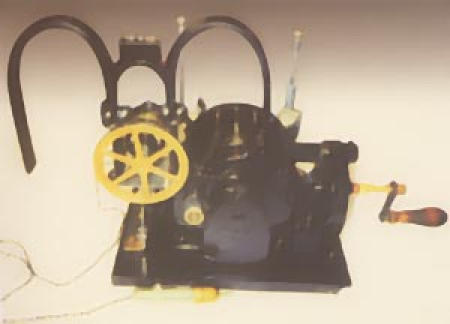
Hyman Pacemaker

In 1930 Albert S. Hyman decided on the need to resuscitate persons whose hearts had entered standstill. He did not distinguish between cardiac arrest and ventricular fibrillation and his published articles did not discuss heart block. He had previously described the use of intracardiac (right atrial) injections of many medications, including epinephrine. Among the techniques he also devised was a machine to produce electricity to be introduced into the heart by a needle plunged through the chest wall, and would cause it to beat. With his brother, an engineer, they developed and patented the "artificial pacemaker" operated by a hand crank and spring motor which turned a magnet (DC current generator) to supply the electricity (Figure 1). The device was used in the New York area and received press coverage, though not acceptance by the medical community. The concept was also criticized as interfering with natural events.

## The 1940s: Hopps Pacemaker-Defibrillator

**Figure 2:**
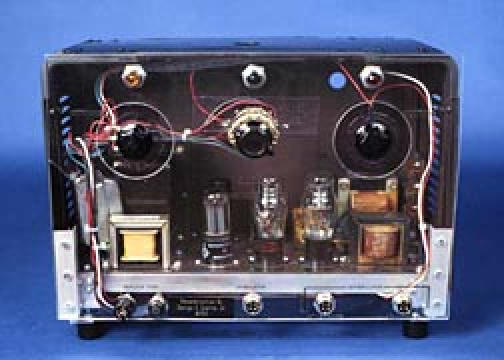
Hopps Pacemaker-Defibrillator

In 1949 Wilfred Bigelow, MD of the University of Toronto and his trainee, John C. Callaghan, MD, began developing hypothermia for use in reducing metabolism and producing bradycardia during which intracardiac procedures could be performed. At a temperature of 21C, asystole occurred. Cardiac contraction could not be restored sufficiently rapidly by re-warming the body, so experimentation with stimulation of the sino-atrial node was begun. An engineer, John A. Hopps, was assigned by the National Research Council of Canada to participate. He designed the first catheter electrode for cardiac stimulation, which was introduced via the right external jugular vein of the experimental animal and a vacuum tube operated external pacemaker (Figure 2). Atrial pacing was readily achieved and control of the cardiac rate was accomplished [2]. The photo seen is a reproduction of the original Hopps Pacemaker-Defibrillator, which was constructed by George Szarka. It is a working model duplicating the original Hopps circuit. The front panel is of clear plastic to allow visualization of the circuit element.

## The 1950s: Zoll Pacemaker

**Figure 3:**
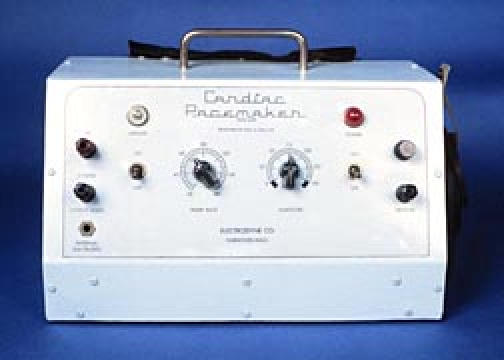
Zoll Pacemaker

In 1950 Paul M. Zoll began work on an external pacemaker which was to stimulate the heart across the closed chest (Figure 3). The pacemaker was line current operated and put out a maximum of approximately 150 volts. Output voltage and stimulation rates were controlled from the front panel of the pacemaker. The electrodes were two one-inch diameter metal discs placed on the right and left sides of the chest, held in place by a rubber strap and making contact via a conductive electrode jelly. By 1952 the first papers concerning the pacemaker were published [3] and revolutionized the concept of resuscitation of the patient with heart block and asystole. Stimulation of an adult required approximately 100 volts, some sufficing with less and others requiring higher voltage. Stimulation was painful and required sedation. Prolonged stimulation produced local skin burns. The longest period of stimulation reported was 11 days. Beginning and end of stimulation was by manual switch operation. By 1956, the need for stimulation via myocardial wires placed during surgery caused the placement of an outlet labeled "internal" and from the output voltage was reduced to one tenth.

For further reading, please visit the Electricity and the Heart Website: http://www.naspe.org/library/electricity_and_the_heart
